# Remote Electrical Stimulation by Means of Implanted Rectifiers

**DOI:** 10.1371/journal.pone.0023456

**Published:** 2011-08-05

**Authors:** Antoni Ivorra

**Affiliations:** Department of Information and Communication Technologies, Universitat Pompeu Fabra, Barcelona, Spain; University of California, Berkeley, United States of America

## Abstract

Miniaturization of active implantable medical devices is currently compromised by the available means for electrically powering them. Most common energy supply techniques for implants – batteries and inductive couplers – comprise bulky parts which, in most cases, are significantly larger than the circuitry they feed. Here, for overcoming such miniaturization bottleneck in the case of implants for electrical stimulation, it is proposed to make those implants act as rectifiers of high frequency bursts supplied by remote electrodes. In this way, low frequency currents will be generated locally around the implant and these low frequency currents will perform stimulation of excitable tissues whereas the high frequency currents will cause only innocuous heating. The present study numerically demonstrates that low frequency currents capable of stimulation can be produced by a miniature device behaving as a diode when high frequency currents, neither capable of thermal damage nor of stimulation, flow through the tissue where the device is implanted. Moreover, experimental evidence is provided by an in vivo proof of concept model consisting of an anesthetized earthworm in which a commercial diode was implanted. With currently available microelectronic techniques, very thin stimulation capsules (diameter <500 µm) deliverable by injection are easily conceivable.

## Introduction

Miniaturization of implantable smart devices for diagnosis and therapeutics is currently compromised by the available means for supplying those devices with electrical energy. Although some ingenious energy harvesting techniques are under research [1], electrochemical batteries and inductive coupling systems are found in the vast majority of implantable electronic devices; and these two electricity sources comprise bulky parts which, in most cases, are significantly larger than the microelectronic circuitry they feed.

In 1991, Loeb et al. introduced a novel paradigm for Functional Electrical Stimulation (FES): they conceived and developed devices for electrical stimulation of excitable tissues that were small enough for being delivered by injection [2]. These devices, later known by the commercial name BION [3], were intended for solving some major constraints of existing techniques for performing multi-site stimulation. The first BION devices had a length of about 16 mm and a diameter of 2 mm and were energized solely by inductive coupling with an external large coil fed by high currents; later devices (BION 3 ABC) have a length of 27 mm and a diameter of 3.3 mm and contain a rechargeable battery that is charged by means of inductive coupling [3]. Quite probably, to date, these are the smallest implantable systems with some sort of medical functionality based on electricity.

Inductive coupling depends on Faraday's law of induction which states that the induced electromotive force (EMF) in any closed circuit is equal to the time rate of change of the magnetic flux through the circuit. Therefore, since the magnetic flux through the circuit is proportional to the area of the surface enclosed by the circuit, then the total area of the circuit generating the EMF, that is, the coil of the implant, is a fundamental parameter for achieving effective inductive coupling between the external magnetic field generator and the implant. BION devices partially overcome this by using a miniature coil with multiple turns and a ferrite core [4]. However, even with multiple turns and a ferrite core, a minimum area (i.e. diameter) is still required for the coil. This is the fundamental reason why BION devices were not made smaller and why it is difficult to conceive devices based on inductive coupling with diameters significantly below 1 mm [5].

**Figure 1:**
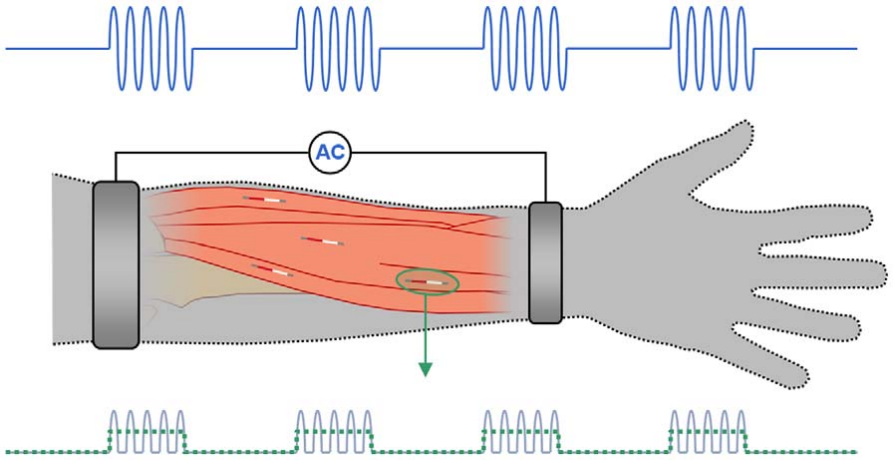
Illustration of a hypothetical use of the method proposed here for a FES application. The capsules, consisting of a very thin diode and two electrodes in its simplest version, would be implanted by injection within the muscles that require stimulation, close to their motor points. Bursts of AC current would be forced to flow through tissues by means of external electrodes so that locally, at the vicinity of the capsules, low frequency current densities capable of stimulation would be produced by rectification.

Here it is proposed an innovative method in order to overcome the miniaturization bottleneck imposed by the existing powering techniques: the implanted devices will act as rectifiers of innocuous high frequency AC currents supplied to the tissue of interest by remote electrodes. In this way, DC or low frequency currents will be generated locally around the implants and these resultant currents will carry out a therapeutic or diagnostic action. For stimulation of excitable tissues, high frequency AC currents can be delivered as bursts so that low frequency pulses are virtually created in the implant. This concept is schematically illustrated in figure 1. The remote electrodes can be external surface electrodes and the AC current feeding them may come from a portable generator.

A key phenomenon for the feasibility of the proposed method is that AC currents with frequencies above 10 kHz hardly cause any sort of stimulation of excitable tissues; their physiological impact is considered to be solely thermal due to the Joule effect. Actually, it is commonly stated that AC currents with frequencies above 10 kHz are incapable of stimulating any excitable tissue regardless of their magnitude [6]. As a matter of fact, AC currents with frequencies above 10 kHz do stimulate excitable tissues but with much higher magnitude excitation thresholds than those of frequencies in the range from 10 Hz to 100 Hz. For instance, it has been measured that current density thresholds for 1 MHz currents are above 50 times those of 100 Hz currents [7]. Therefore, in principle, if the implants can generate current densities with a low frequency (10 to 100 Hz) component that has a magnitude significantly above 1/50 of the high frequency current density applied by the remote electrodes, it will be possible to perform local stimulation without causing any undesired stimulation of neighbor tissues.

A constant AC current flowing the tissues will cause DC currents around the rectifying implants whereas amplitude modulated AC currents will cause currents around the implants with some frequency contents. In particular, here it is proposed to deliver the AC currents (100 kHz or higher) as bursts with repetition rates from 1 Hz to 100 Hz so that virtual pulses of equivalent frequencies are created. Since pulsed currents at those low frequencies are much more effective for stimulation than DC currents, delivery of AC burst will be more energetically efficient and will result in less Joule heating than constant AC current.

As first step for validating the usefulness of the proposed method, the present study first numerically verifies that it is possible to obtain local low frequency current densities with a magnitude well above 1/50 of that of the high frequency current density applied by remote electrodes. Moreover, it is verified that those low frequency current magnitudes, and related voltages, can be in the same order of those applied by BION stimulators and that the remotely applied high frequency current does not cause a temperature increase capable of tissue damage. In addition to that, experimental evidence is reported from an *in vivo* proof of concept model consisting of an anesthetized earthworm in which a commercial diode was implanted.

## Methods

### Computer simulation

A Finite Element Method (FEM) simulation has been carried out in order to demonstrate that an implanted diode in a resistive medium can produce local current densities with significant mean values (i.e. DC component) when an innocuous AC current flows uniformly through that medium.

The FEM software used for this simulation was COMSOL Multiphysics 4.0a (COMSOL AB, Stockholm, Sweden) and the so called Physics modules were “Electric Currents”, “Electrical Circuit” and “PDE (c)” for a bi-dimensional geometry with axial symmetry.

**Figure 2:**
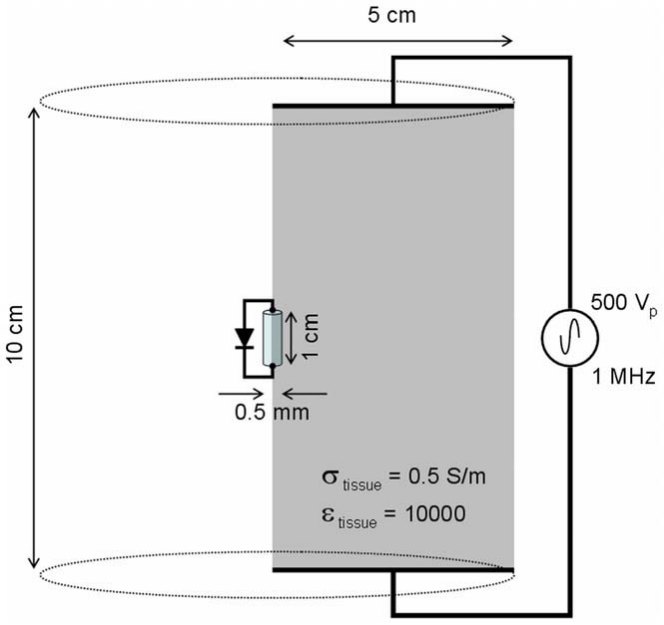
Geometry of the model employed for the FEM simulation. It is solved with axial symmetry around the axis of the implant (drawing not to scale).

The geometrical features of the model, together with other key parameters, are depicted in figure 2. The implant consists of a cylinder with a length of 10 mm and a diameter of 0.5 mm. The material of the cylinder is selected to be an insulator (conductivity, σ, = 1×10^−6^ S/m) but it behaves as a diode between its top and bottom surfaces, which act as the electrodes of the implant. Modeling of the diode is possible thanks to the SPICE capabilities of the “Electric Circuits” Physics module of COMSOL Multiphysics. The parameters of diode are selected so that it behaves as a common Schottky diode with a low forward voltage (saturation current, I_F_, = 1×10^−5^ A; series resistance, R_S_, = 0.5 Ω). The conductivity of the medium is set to 0.5 S/m and its relative permittivity to 1000 which are values similar to those reported for experimental measurements of muscle dielectric properties at 1 MHz [8]. The FEM contains 4111 triangular elements.

A single cycle of a 1 MHz sinusoidal voltage is applied between top and down surfaces of the cylinder that represents the tissue. The amplitude of that voltage is adjusted to 500 volts, which produces an electric field of 5000 V/m, as it was found that such amplitude produces an average voltage drop between the two implant electrodes of about 16 V which is very close to the maximum voltage deliverable by BION devices (voltage compliance = 17 V) [3].

### Thermal analysis

Assuming that the sinusoidal applied field will have a maximum amplitude of 50 V/cm together with BION maximum values for the other stimulation parameters (repetition rate up to 50 pulses per second and pulse duration up to 250 µs) [3], now it is possible to compute the maximum temperature that will be reached in a plausible scenario for the method: continuous stimulation of skeletal muscle.

For this analysis, here it is employed the so called *Pennes Bioheat Equation* in which Joule heating is integrated:
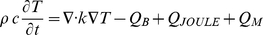
(1)where *T* is the temperature, *ρ* is the mass density of the tissue, *c* is its specific heat, *k* is its thermal conductivity, *Q_M_* is the metabolically generated heat, *Q_JOULE_* is the heat generated by the electric field and *Q_B_* is the heat removed by blood perfusion. Since Joule heating is generated uniformly in the tissue, it is reasonable to disregard the heat diffusion term and, since here only temperature rise due to Joule heating is of interest, the metabolic contribution can also be disregarded so that equation 1 is transformed into:
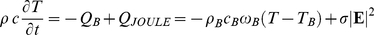
(2)where *ρ_B_* is the mass density of the blood, *c_B_* is its specific heat, *ω_B_* is the blood perfusion rate in the tissue, *T_B_* is the blood temperature, *σ* is the tissue electrical conductivity and |**E**| is the root mean square value (RMS) of the electric field.

The final temperature for the differential equation 2 is:
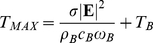
(3)Then, since the sinusoidal electric field will not be applied continuously but as bursts with a maximum duration of 250 µs and a repetition rate of up to 50 pulses per second, Joule heating has to be scaled down to:
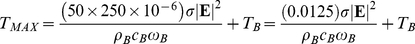
(4)With realistic values from literature for the biophysical parameters (*ρ_B_* = 1060 kg/m^3^, *c_B_* = 3960 J/(kg.K), *ω_B_* = 0.0085 s^−1^, *T_B_* = 37°C) [9] (*σ* = 0.5 S/m) [8], the reached temperature for an electric field with a RMS value of 3500 V/m (|**E**|_P_ = 5000 V/m) is 39.1°C, that is, a temperature increment of 2.1°C

### In vivo proof of concept

**Figure 3:**
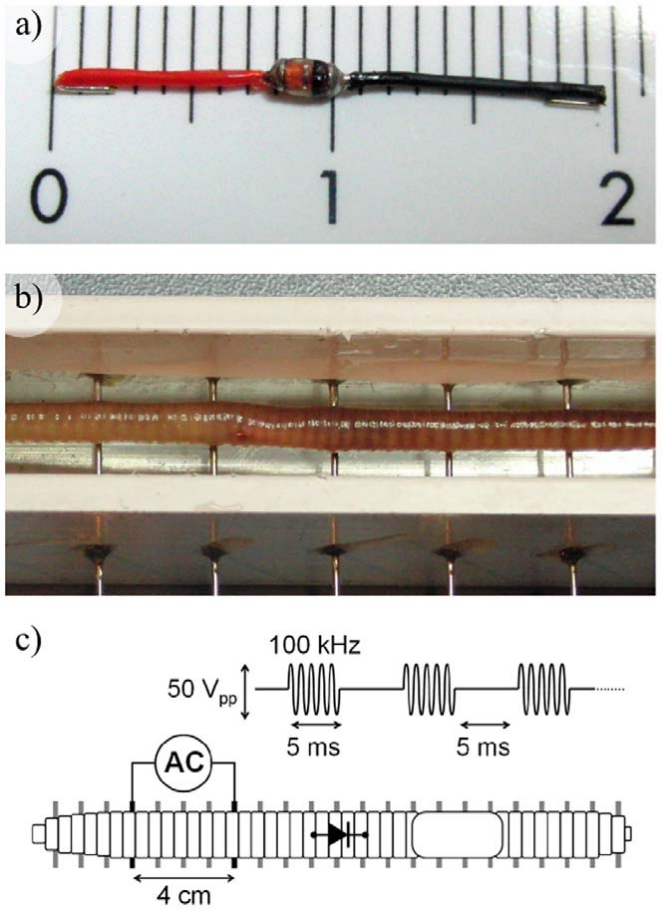
Proof of concept model. a) Picture of the rectifier implant developed for proof of concept; two wires acting as electrodes connected to a Schottky diode. b) The implant is barely noticeable once inserted in the earthworm (lying on an array of horizontal needles acting as electrodes). c) Schematic representation of the experimental setup (First, the AC bursts are applied between two electrodes that do not flank the region in which the implant is located and no movement is observable).

A device consisting of a Schottky diode and two electrodes (Figure 3.a) was implemented for implantation in an anesthetized earthworm. The Schottky diode (model MCL103B-TR by Vishay Semiconductor GmbH, Heilbronn, Germany) has a length of 2 mm and a diameter of 1.2 mm. Thin insulated wires (models 100-30R and 100-30BK by Pro Power distributed by Farnell Components, S.L., Cornella, Spain) were soldered to the cathode and anode of the diode so that a thin device with a length of about 24 mm was obtained. The distal 2 mm of each wire were exposed for forming the electrodes of the device and were bent inwards so that the device length was reduced to approximately 20 mm. Then the diode and the solder joints were insulated with a varnish (model MR8008B by Electrolube distributed by Farnell Components, S.L., Cornella, Spain).

Live earthworms (Lumbricus sp.) were acquired from a local bait shop (La Pesca “Antigua Casa La Viuda”, C/General Castaños 6, Barcelona) and kept in a fridge at 10°C for a few days before the experiment. Prior to diode implantation, the earthworm was anesthetized by immersing it for 15 minutes in a 0.2% tap water solution of Chlorobutanol (1,1,1-Trichloro-2-Methyl-2-Methyl-2-Propanol) (Catalogue number 112054-50 G by Sigma-Aldrich Co., St. Louis, MO, USA). Then the earthworm dorsal skin was punctured with a 22 G needle and the device was gently pushed into the body of the earthworm (barely noticeable in figure 3.b). The earthworm was then laid on a parallel array of horizontal stainless needles separated at constant distance of 10 mm; this experimental electrode setup is almost identical to the one proposed in [10].

A function generator (model AFG3022 by Tektronix, Inc., Beaverton, OR, USA) and a custom developed power amplifier were employed for delivering bursts of 100 kHz with amplitude 25 volts, period 10 ms and duty cycle 50%. Those bursts were applied between two needles of the parallel array on which the earthworm was laying. The distance between the needles was always set to 4 cm so that a field of roughly 6 V/cm developed in the region of the earthworm flanked by the active needles. First it was tried to deliver the signal between needles that did bound the region in which the implanted device was located (figure 3c). Careful observations were made in order to rule out any movement or spasm. Then, the signal was applied between needles that bounded the region in which the device was implanted and an immediate and strong spasm was observed. An execution of the experiment is provided as a supporting video to this document (Video S1).

## Results

### Computer simulation results

**Figure 4:**
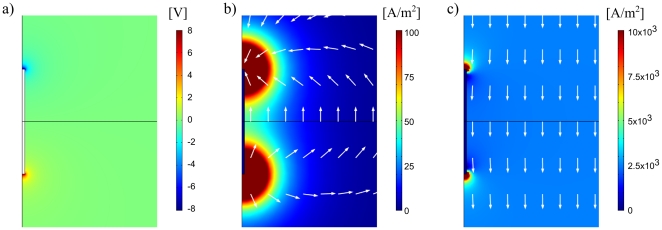
FEM simulation results. Close up of the implant region when a single period of a 1 MHz sinusoidal signal with amplitude 500 V is applied. a) Average (i.e. DC) voltage for the whole period. b) Average (i.e. DC) current density. White arrows indicate current density direction, not magnitude. c) Current density amplitude.

Delivery of a sinusoidal electric field of 50 V/cm to the region where the 10 mm rectifying implant is located produces an average (i.e. DC) voltage drop between the implant electrodes of about 16 V (figure 4.a). Note that this value closely corresponds to the average value for half-wave rectification of sinusoidal signals (0.318×V_P_) taking V_P_ as the product of |**E**|_PEAK_ by the electrode distance (i.e. implant length). Therefore, without increasing the applied sinusoidal voltage, it would be possible to increase the average voltage drop at the implant by elongating it or by using signals that produce larger averages (e.g. a square signal results in an average voltage after rectification of 0.5×V_P_).

The average voltage drop caused between the implant electrodes obviously induces an average current flowing through the tissue (∼6 mA). Current density map corresponding to such current is displayed in figure 4.b. It is important to note that a significant region around the electrodes experiences current densities above 50 A/m^2^ and that this value corresponds to 1/50 of the peak current density caused by the sinusoidal voltage (figure 4.c) which is 2500 A/m^2^ (|**J**| = *σ*|**E**| = 0.5 [S/m]×5000 [V/m]). Therefore, as hypothesized, it will be possible to generate current densities with a low frequency component that has a magnitude significantly above 1/50 of the high frequency current density applied by the remote electrodes, which is the theoretical condition for performing local stimulation without causing any undesired stimulation of tissues through which the high frequency current flows.

### Thermal analysis results

As already mentioned in the methods section, conditions that correspond to the maximum stimulation capabilities of BION devices (voltage compliance = 16 V, pulse duration = 250 µs and pulse repetition frequency = 50 pulses per second) would yield an increase in temperature of only about 2°C in the case of skeletal muscle if the AC bursts were applied continuously. Due to differences in electrical conductivity and thermal properties, other tissues could result in lower or higher increases of temperature. However, temperature increases that could result in tissue damage are difficult to conceive. Moreover, it is worth noting that typical conditions for BION devices are in the order of less than 7 V (4 mA in a medium with a conductivity of 0.2 S/m) and 4 pulses of 200 µs per second [11], which would yield an insignificant increase in temperature of about 0.02°C (simulations and calculations not reported here).

In addition, it needs to be mentioned that continuous (i.e. permanent) stimulation is not always required. Depending on the application, stimulation episodes of a few minutes may be preceded and followed by quiet episodes of minutes and hours.

### In vivo proof of concept results

A video (Video S1) is provided as supporting information showing an execution of the experiment detailed in the methods section. Observations are already mentioned in the methods section.

## Discussion

Here it has been shown *in vivo* that the proposed method indeed can generate virtual current pulses able to stimulate muscles when a train of high frequency bursts of current (*per se* incapable of stimulation) is applied in the region where the implant is located. The simple implant configuration used here, consisting of a diode and two electrodes, will allow applications such as temporary or experimental FES systems and therapeutic techniques in which DC currents are required such as local electrochemical ablation of tissues [12], wound healing [13] [14] and bone fracture repair [15]. For other applications it will be required to embed more sophisticated electronics within the module. For instance, in order to minimize electrochemical damage to the electrodes and tissues in FES clinical applications or in electrical stimulation for pain relief [16] or management of some Central Nervous System (CNS) diseases such as Parkinson's disease [17], it will be convenient to include a mechanism for switching the polarity of the rectifier so that charge balance can be achieved [18]. In addition to that, other electronic functions that will be very interesting to embed within the module are: demodulation of code signals contained within the AC bursts for addressing specific implants and load modulation for transmitting data towards the external systems. Regarding this last aspect it is worth noting that the regulated load used for data transmission will not be contained within the implant: the regulated load will actually be the living tissues around the implant; that is, the implant will act as the switch for such tissue load. Hence, by removing the need for an internal load, further miniaturization will be possible. With these advanced features embedded within the implant (addressing and data transmission) it is easy to conceive interesting applications of the method such as multi-channel FES systems, recording of action potentials or monitoring of physiological parameters measurable with sensors. Since skull impedance is low at high frequencies [19], it is even possible to speculate about more sophisticated applications such as multi-site electrical stimulation of the brain cortex for restoration of vision or to use LEDs rather than standard diodes for performing stimulation of genetically modified brain cells [20]. From a clinical point of view, a very interesting feature of the method is its compatibility with magnetic resonance imaging (MRI) techniques as no ferromagnetic materials are strictly required for the implants.

In addition to medical applications, it is possible to envisage other uses of the proposed method such as implantable identification capsules. Besides miniaturization, these capsules based on the method may offer some advantages over current implantable identification devices based on inductive or near-field coupling: by requiring direct contact between the user and the external reader device, such capsules will be less prone to the so called *man-in-the-middle attack* and it will not be possible to employ them as involuntary tracking devices.

Currently, smallest packaged commercial diodes are about 0.6 mm×0.3 mm×0.3 mm (0201 SMD package), and complete systems as complex as RFID tags are embedded in integrated circuits with an area of only 0.4 mm×0.4 mm [21], therefore, based on the proposed method, smart stimulation capsules with a diameter below 0.5 mm are easily conceivable with existing microelectronic techniques. Moreover, it has to be taken into account that the two ends of the implant can consist of very thin (<100 µm) flexible wires. Hence the implants based on the method may look like very short pieces of thread rather than thick and rigid capsules. In addition, the method could benefit from current efforts in miniaturizing electronic components down to the nanometer scale.

It must be mentioned that a drawback of the proposed method is its extremely poor energy efficiency. In the simulated case, for a 10 ms pulse per second application, delivery of only 960 µW of DC power at the implant (16 V, 6 mA) requires 49 W as remote high frequency power (500 V_P_, 19.6 A_P_), which implies an efficiency below 0.002%. Nevertheless, this does not imply that portable equipment will be ruled out. If a more realistic stimulation case is analyzed such as the one mentioned earlier (stimulation voltage = 7 V and 4 pulses of 200 µs per second) then it is found that less than 1 W would be required, which can be supplied for many hours with portable batteries.

Although the presented method has some functional similarities with the so called Stimulus Router System (SRS) proposed by Gan and Prochazka [22], in which low frequency stimuli from external electrodes are conducted through subcutaneous electrodes and wires to the motor points where stimuli are focused, it must be recognized that the method proposed here is based on completely different phenomena and that it very significantly facilitates the implantation of the systems, particularly for multi-site stimulation.

### Conclusions

It has been demonstrated numerically that low frequency currents capable of stimulation can be produced by a miniature device behaving as a diode when high frequency currents, neither capable of thermal damage nor of stimulation, flow through the tissue where the device is implanted. Moreover, experimental evidence is provided by an *in vivo* proof of concept model consisting of an anesthetized earthworm in which a commercial diode was implanted.

The proposed innovative method may be foundational to a broad range of new developments in the field of implantable medical devices, ranging from wound healing to nerve stimulation for pain relief. In addition, other non-medical devices could also emerge such as implantable identification capsules.

## Supporting Information

Video S1 **In vivo proof of concept model.**
(WMV)
